# Estimating the efficiency of primary health care services and its determinants: evidence from provincial panel data in China

**DOI:** 10.3389/fpubh.2023.1173197

**Published:** 2023-06-15

**Authors:** Zhe Zhao, Silai Dong, Jiahe Wang, Qingzhi Jiang

**Affiliations:** ^1^School of Public Administration, Huazhong Agricultural University, Wuhan, China; ^2^Asia-Pacific Institute of Ageing Studies, Lingnan University, Tuen Mun, Hong Kong SAR, China

**Keywords:** efficiency, primary health care services, determinants, financial support, social health insurance

## Abstract

**Background:**

The efficiency of primary health care services is drawing increased attention worldwide, especially in developing countries. Health care reform in China has moved into the ‘deep water zone’ phase and is facing the dilemma of inefficiency in primary health care services, which is a critical challenge for universal health coverage.

**Methods:**

In this study, we estimate the efficiency of primary health care services in China and its determinants. A combination of a super-SBM (Slack-Based Measure) model, a Malmquist productivity index model and a Tobit model is used to study provincial panel data, and the results demonstrate the inefficiency of primary health care services in China and the variations in efficiency values between regions.

**Results:**

Over time, the productivity of primary health care services shows a decreasing trend, mainly due to slowing technology change. Financial support is needed to improve the efficiency of primary health care services, but it is worth noting that existing social health insurance coverage decreases efficiency, while economic development, urbanization and education also have a significant impact.

**Conclusion:**

The findings suggest that increasing financial support should remain a priority in developing countries but that reasonable reimbursement design, appropriate payment methods and comprehensive supporting social health insurance policies are key to the next step of reform.

## 1. Introduction

According to the World Health Organization, the fundamental goal of global health system reform is to achieve universal health coverage, and countries around the world continue to reform their health care systems with the aim of improving service structures, processes and outcomes (1). Primary health care (PHC) is widely seen as the backbone of a national health care system that provides comprehensive services to the population, but both developed and developing countries have been caught in the dilemma of inefficiency in PHC services. Efficiency improvements are critical for reducing wasted resources and achieving sustainable health outcomes (2), especially in developing nations.

Efficiency is a measure of the amount of output in relation to a given level of input (3), and technical efficiency (TE) is often used to represent the efficiency of health care services. Whether greater input improves efficiency is unclear; although some studies have noted that insufficient input can limit efficiency (4), increased input does not itself necessarily lead to increased efficiency. An estimation is therefore beneficial, especially for developing countries, when PHC reform reaches the point at which the impact of increased input becomes unclear and governments begin looking for solid evidence to improve their existing reform agenda. Existing research has mostly focused on developed countries at the institutional (5–7) and system levels (8–10), but much less attention has been paid specifically to PHC services (11, 12), and more empirical research is needed to support the estimation of PHC service efficiency in developing countries in particular.

The next most pressing question is that of which determinants are associated with efficiency, which this study also aims to answer. Recent studies have found that socio-economic factors, including population, urbanization, GDP *per capita* and education, have an effect on the efficiency of health care systems (13–15). Two policy tools—increased financial support and social health insurance coverage—are also often used in an attempt to reform health and primary care, but whether they improve the efficiency of PHC services is unclear. A study of 165 countries, for example, found a positive correlation between the share of health expenditure in the public budget and the performance of the health system (16), but evidence from emerging and developing economies shows that simply increasing public spending can reduce efficiency if done so without sufficient supervision (17). Findings from 21 OECD countries indicate that offering insurance coverage to a larger percentage of the population can facilitate health system efficiency (18), but reforms in the United States have shown that increased insurance coverage can also decrease the TE of health care delivery (19). However, as previously noted, while there is a wealth of research on developed countries, detailed evidence in developing countries is still limited, especially for PHC services, and this study seeks to narrow this knowledge gap.

As the largest developing country in the world, China was praised between the 1950s and 1970s as a successful example of a nation addressing its health care issues. The foundation of the PHC system was laid by a community-based rural health insurance programme (Cooperative Medical System; CMS), ‘barefoot doctors’ and a three-tier delivery system (county hospitals, township health centers and village clinics). Three social insurance schemes—the Government Insurance Scheme, Labor Insurance Scheme and CMS—covered almost the entire population, and health reform initiatives that started in 1978 have since gone through three phases (20). During the first stage, China transitioned from a planned economy to a socialist market economy, and the government adopted a *laissez-faire* market for health care to fund and deliver health services. PHC become the least developed and most vulnerable part of the health care system (21), and the health insurance system began to collapse with the decrease in public provision and the dissolution of the CMS. The beginning of the second stage of health care reform was marked by the introduction of the Urban Employees’ Basic Medical Insurance (UEBMI) for formally employed workers in 1998, which was followed by the New Rural Cooperative Medical Scheme (NRCMS) for rural residents in 2003 and the Urban Residents’ Basic Medical Insurance (URBMI) for unemployed urban residents in 2007. In 2009, the third stage of health system reform was launched, which, in terms of scale and scope, is one of the largest health policy interventions in modern history. The plan specifically identifies five targets, one of which is restoring China’s once enviable PHC system, which will lead to the rebuilding of a well-structured delivery system.

**Figure 1:**
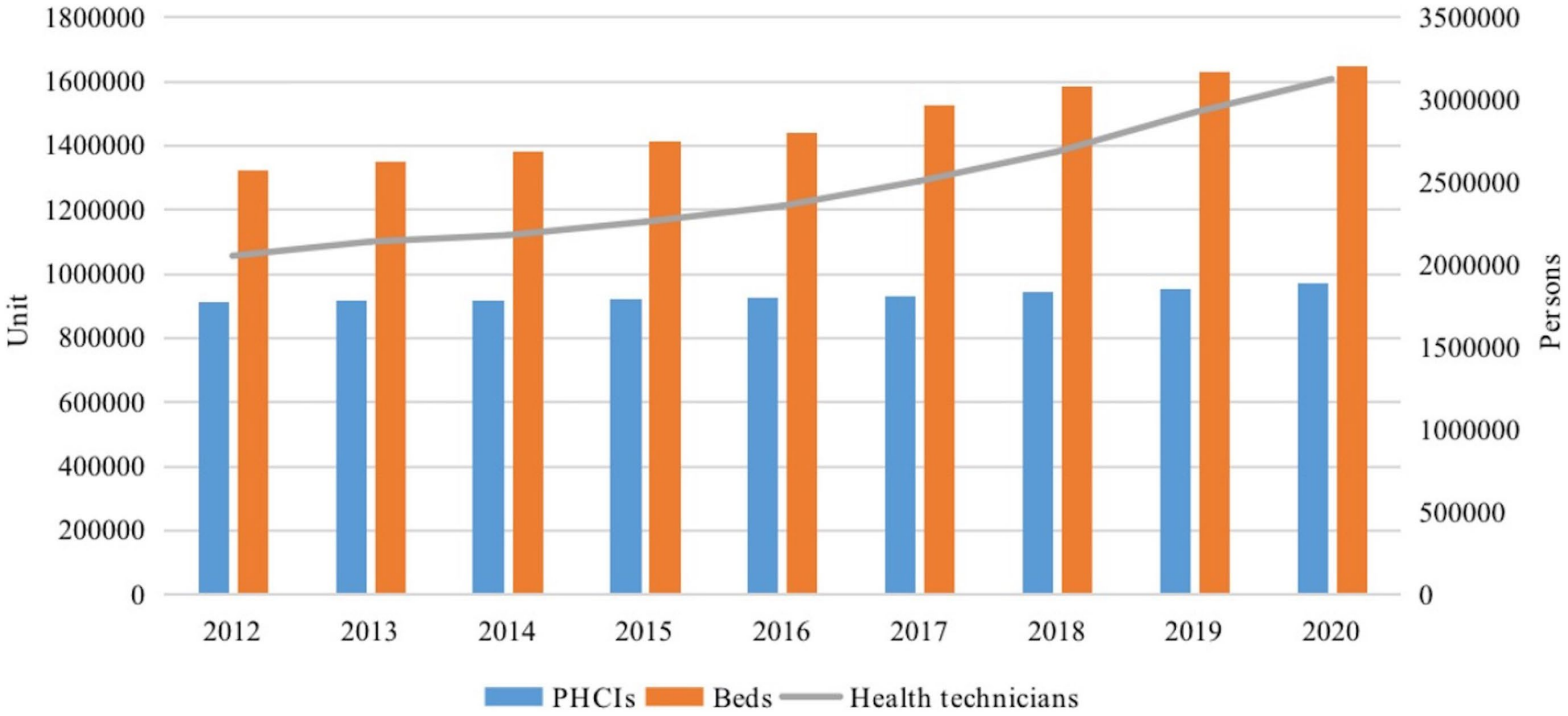
Number of PHCIs, beds and health technicians in PHCIs, 2012–2020.Source: China Health Statistical Yearbook (2013–2021).

Primary care facilities are a crucial component in the system of health services and health care provision. China’s PHC system consists of community health service centers (stations), sub-district health centers, township health centers, village clinics, outpatient departments and infirmaries, which serve as the first line of defense against health inequities. From 2012 to 2020, the financial subsidy income of primary health care institutions (PHCIs) increased by 176% in absolute terms and had grown to 33.1% of the total income of PHCIs by 2020. Meanwhile, a huge amount of money was invested to bring more facilities, equipment and human resources to the PHC system (see Figure 1), and continuous growth was observed in PHCIs, beds and especially health technicians, which increased by a total of 52.2%. Great efforts have also been made to implement health care alliances, and a series of policy measures have been introduced to improve the quality of services in order to encourage residents to use PHC facilities. Although the number of visits to PHCIs is increasing, the ratio of PHC visits to the total number of medical visits has remained between 50 and 60%, and the efficiency of PHC services in China still faces serious challenges.

The main goals of the study were to (1) estimate the efficiency of PHC services in China and their productivity changes; (2) analyze the determinants of the efficiency of PHC services in mainland China; and (3) provide policy implications for developing countries and for improving the efficiency of PHC services.

## 2. Materials and methods

We used a super-SBM model to measure TE, a Malmquist index model to measure productivity changes and a Tobit model to analyze the determinants of efficiency. These methods have been widely used to estimate the efficiency of health performance in both developed (22) and developing (23, 24) countries.

### 2.1. Measuring technical efficiency

#### 2.1.1. Super-SBM model

TE reflects the capacity to provide health care services by combining and utilizing the available production factors (25). Tone (26) proposed a non-radial, non-angular SBM model that includes slack variables; since a single SBM model has an optimal efficiency evaluation of 1, there are often multiple decision-making units (DMU) with an efficiency value of 1, making it impossible to perform a comparative ranking. Tone (27) therefore proposed the super-SBM model, which derives efficiency values that can be greater than or equal to 1, effectively solving the problem of the relative efficiency ranking of DMUs. The specific formula is


(1) $$\min\sigma =\frac{1+\frac{1}{m}\overset{m}{\underset{i=1}{\sum }}\bar{s_{i}}/x_{ik}}{1-\frac{1}{q}\overset{q}{\underset{r=1}{\sum }}\overset{+}{s_{r}}/y_{rk}}$$

which is subject to


(2) $$\overset{n}{\underset{j=1,j\neq k}{\sum }}x_{ij}\lambda_{j}-\bar{s_{i}}\leq x_{ik}$$

(3) $$\overset{n}{\underset{j=1,j\neq k}{\sum }}y_{rj}\lambda_{j}+\overset{+}{s_{r}}\geq y_{rk}$$

(4) $$\lambda ,\;\;\;s^{-},\;\;\;s^{+}\geq 0$$

(5) r=1,2,…q,i=1,2,…m,j=1,2,…n

where *σ* is the TE value; *x* and *y* are the observed values of DMU inputs and outputs, respectively; *s^−^* and *s^+^* represent the input and output slacks, respectively, for the DMU under evaluation; and *λ* is the weight coefficient of the reference DMU. The super-SBM model assumes a constant return to scale (CRS). This study extends the super-SBM models to the variable return to scale (VRS) case by limiting


$$\overset{n}{\underset{j=1,j\neq k}{\sum }}\lambda_{j}=1$$

for Eq. 5.

### 2.2. Measuring the productivity change

#### 2.2.1. Malmquist productivity index model

The Malmquist productivity index (MPI) model is used to measure the dynamic changes in total factor productivity (TFP) in DMUs over time. TFP is divided into technology change (TC), which reflects the movement of the production frontier surface from period *t* to period *t* + 1, and technical efficiency change (TEC), which represents the rate at which the DMU catches up with the production possibility frontier from period *t* to period *t* + 1 and which can be further decomposed into pure technical efficiency change (PEC) and scale efficiency change (SEC). The formula is


(6) MPI=TFP=TEC×TC=PEC×SEC×TC

We can see the TFP from year *t* to year *t* + 1 in Eq. 7:


(7) $$\begin{array}{c} MPI=TFP=\left(x^{t+1},\;y^{t+1};\;x^{t},\;y^{t}\right)=\sqrt{\frac{D^{t+1}\left(x^{t},\;y^{t}\right)}{D^{t+1}\left(x^{t+1},\;y^{t+1}\right)}\times \frac{D^{t}\left(x^{t},\;y^{t}\right)}{D^{t}\left(x^{t+1},\;y^{t+1}\right)}}\\ =\frac{D^{t}\left(x^{t},\;y^{t}\right)}{D^{t+1}\left(x^{t+1},\;y^{t+1}\right)}\sqrt{\frac{D^{t+1}\left(x^{t},\;y^{t}\right)}{D^{t}\left(x^{t},\;y^{t}\right)}\times \frac{D^{t+1}\left(x^{t+1},\;y^{t+1}\right)}{D^{t}\left(x^{t+1},\;y^{t+1}\right)}} \end{array}$$

where *x^t^* and *y^t^* represent the input and output indicators, respectively, in period *t*; *x*^*t* + 1^ and *y*^*t* + 1^ represent the input and output indicators, respectively, in period *t* + 1; and *D^t^* and *D*^*t* + 1^ represent the relative efficiency of DMU in periods *t* and *t* + 1, respectively. As the technical efficiency change tends toward 1 (TEC > 1), the DMU approaches the production frontier and the improvement of TE increases, and vice versa; as the technology change tends toward 1 (TC > 1), the production frontier moves forward with technology progress or innovation, and vice versa; and as the TFP tends toward 1, the DMU’s dynamic efficiency in a certain period increases, and vice versa.

### 2.3. Measuring the technical efficiency determinants

#### 2.3.1. Tobit model

Along with the effects produced by the input and output indicators, other factors can impact the efficiency of PHC services. Using the TE value calculated by the super-SBM model above as the dependent variable and the influencing factors as the independent variables, a regression model can be created that uses the coefficients of the independent variables to determine the direction and intensity of the determinants of efficiency. Tobit regression analysis is a dependent variable constrained model, and the regression model form can be written as


(8) $$Y_{i}=\alpha +\overset{n}{\underset{i=1}{\sum }}\;\beta_{i}X_{i}+\varepsilon ,\;\;\;i=1,2,\ldots ,n$$

where *Y_i_* is the TE value, *X_i_* is the independent variable, *β_i_* is the coefficient to be estimated and *ε* is the random perturbation term.

### 2.4. Data

Data for the indicators in this study were obtained from the China Health Statistical Yearbook (2013–2021) and China Statistical Yearbook (2013–2021), covering the study period from 2012 to 2020. The research subjects were 31 provincial units in mainland China: the eastern region, comprising Beijing, Tianjin, Hebei, Liaoning, Shanghai, Jiangsu, Zhejiang, Fujian, Shandong, Guangdong and Hainan; the central region, comprising Shanxi, Jilin, Heilongjiang, Anhui, Jiangxi, Henan, Hubei and Hunan; and the western region, comprising Inner Mongolia, Chongqing, Guangxi, Sichuan, Guizhou, Yunnan, Shaanxi, Gansu, Qinghai, Ningxia, Tibet and Xinjiang.

#### 2.4.1. Inputs and outputs

In previous studies, input indicators have usually been classified into three categories—capacity-related, labor-related and expense-related—while outputs have usually been classified into activity-related and quality-related, although most studies have used the former rather than the latter (28). In China, input indicators are generally operationalized as the numbers of institutions, beds, health technicians and units of equipment and output indicators as the numbers of visits and inpatient admissions and the bed occupancy rate (29–31). In addition to these direct indicators, some scholars have also used indirect indicators, such as mortality rate and life expectancy (32), but these apply to the national health system rather than to the PHC system specifically and are not direct outcomes of health care, being influenced by other factors, such as ethnicity and diet. Given that the focus of this study is the PHC system and considering data availability, we prefer to use direct indicators: the numbers of PHCIs, beds in PHCIs and health technicians in PHCIs as inputs and the numbers of visits to PHCIs and inpatient admissions to PHCIs as outputs (see Table 1).

**Table 1:** Definitions of inputs and outputs.

Type	Name	Definition
Inputs	Number of PHCIs	PHCIs include community health service centers (stations), sub-district health centers, township health centers, village clinics, outpatient departments and infirmaries.
	Number of beds in PHCIs	Beds include regular beds, simple beds, monitoring beds, extra beds for more than half a year, beds being disinfected and repaired and beds disabled due to expansion or overhaul.
	Number of health technicians in PHCIs	Health technicians include licensed doctors, licensed assistant doctors, registered nurses, pharmacists, laboratory physicians, radiologists and other medical professionals.
Outputs	Number of visits to PHCIs	Number of visits includes outpatient, emergency, single health examination and health consultation visits.
	Number of inpatient admissions to PHCIs	Number of inpatient admissions refers to people who have been admitted to the hospital with the consent of the outpatient physician.

#### 2.4.2. Determinants of technical efficiency

According to some studies, insufficient government investment and a lack of health insurance coverage are the main causes of extreme inefficiency in China’s health care system (33, 34), and we therefore use financial support and social health insurance coverage as independent variables and estimate their relationship with PHC services efficiency. Financial support represents the national financial investment in the health sector, which reflects the importance given to the health care system, and social health insurance coverage reflects the financial protection of the population. We also control for other factors that may influence TE: population density, urbanization, education and GDP *per capita*. The operationalization of the determinants and the hypotheses made regarding the directions of the effects are presented in Table 2.

**Table 2:** Determinants and hypotheses.

Type	Name	Hypothesis	Operationalization
Independent variables	Financial support	Positive (+)	Financial support is measured by the share of health expenditures in public budgets
	Social health insurance coverage	Positive (+)	Social health insurance coverage is measured by dividing number of participants at the end of the year by the total population at the end of the year
Control variables	Population density	Positive (+)	Population density is measured by dividing the population by the area
	Urbanization	Positive (+)	The level of urbanization is measured by the ratio of the urban population to the total population
	Education	Negative (−)	The level of education is measured by the ratio of the illiterate population to the total population aged 15 and above
	GDP *per capita*	Positive (+)	The level of *per capita* GDP is measured by dividing the gross domestic product by the population

## 3. Results

### 3.1. Descriptive statistics

As shown in Table 3, for inputs, there have been continuous increases in institutions, beds and health technicians, with the largest percentage increase from 2012 to 2020 being health technicians, at 52.3%. For outputs, however, the visits and inpatient admissions grew unsteadily from 2012 to 2019, with a significant decrease in 2020. Overall, the growth in outputs was modest, lagging far behind the increase in inputs. It is worth noting that the significant reduction in the number of visits and inpatient admissions was at the start of the COVID-19 pandemic and may be a short-term phenomenon that will reverse as the prevention and control situation improves.

**Table 3:** Descriptive statistics for inputs and outputs.

Indicators	2012	2013	2014	2015	2016	2017	2018	2019	2020
PHCIs	912620	915368	917335	920770	926518	933024	943639	954390	970036
Beds	1324270	1349908	1381197	1413842	1441940	1528528	1583587	1631132	1649384
Health technicians	2051751	2137623	2176823	2257701	2354430	2505174	2682983	2920999	3123955
Visits	410920	432430	436395	434193	436664	442892	440631	453087	411615
Inpatient admissions	4253	4300	4094	4036	4165	4450	4376	4295	3707

### 3.2. Technical efficiency

**Figure 2:**
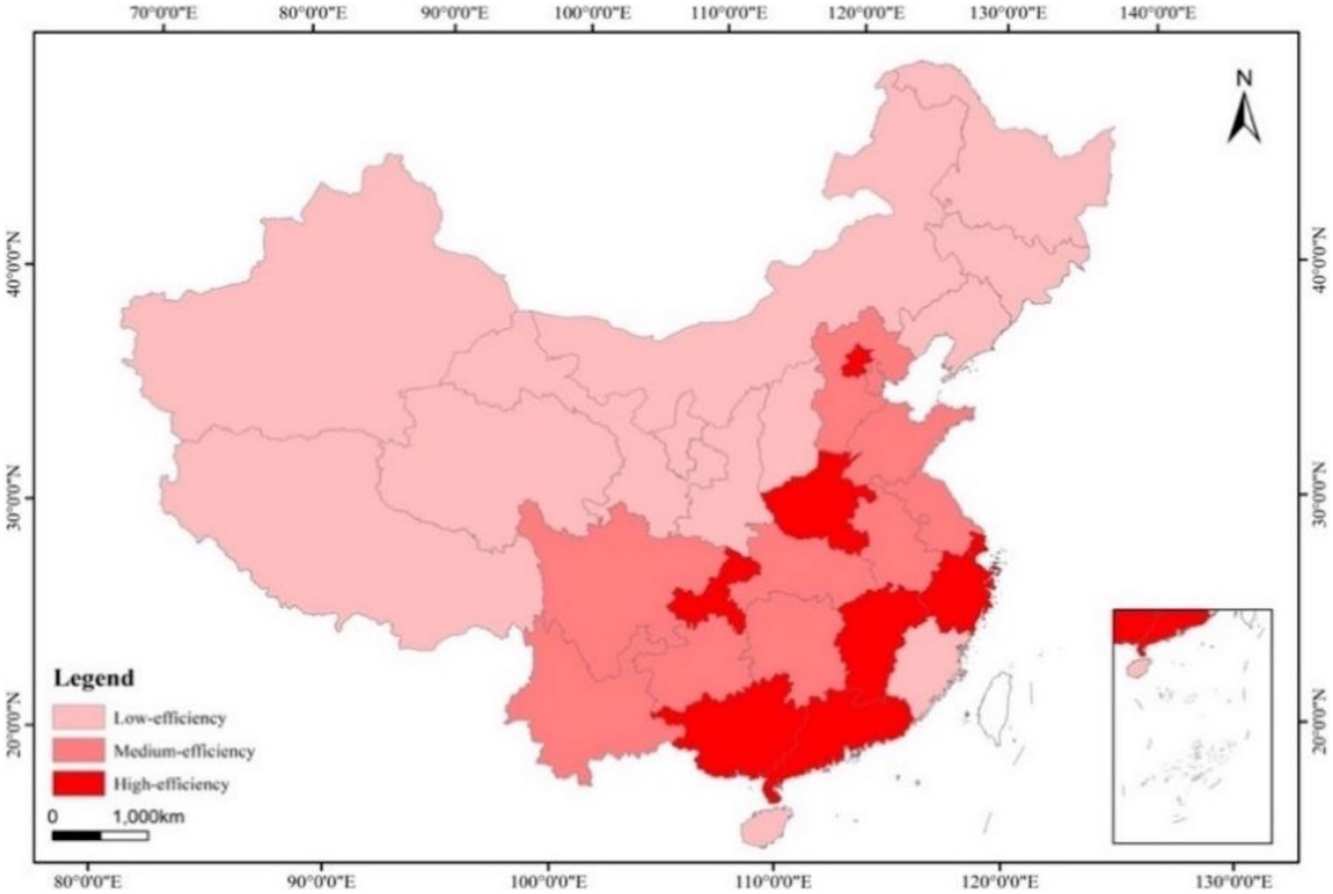
Spatial distribution of primary health care service efficiency.

As shown in Table 4, from 2012 to 2020, the average TE of all 31 provincial units was 0.813 (less than 1), demonstrating the overall inefficiency of PHC services. Based on international studies (35, 36), we divided the efficiency value (EV) into three grades to reflect the spatial distribution characteristics of the efficiency more clearly and intuitively: high-efficiency (EV ≥ 1.0), medium-efficiency (0.8 ≤ EV < 1.0) and low-efficiency (EV < 0.8). ArcGIS10.2 software was used to draw a spatial distribution map (see Figure 2), which shows that half of the high-efficiency provinces are in the eastern region, with the most efficient being Shanghai. The low-efficiency provinces are primarily concentrated in the western region, of which Shanxi has the lowest efficiency.

**Table 4:** Technical efficiency values.

	Province	2012	2013	2014	2015	2016	2017	2018	2019	2020	Average
East	Beijing	1.107	1.141	1.117	1.104	1.125	1.135	1.140	1.155	1.092	1.124
	Tianjin	0.910	1.023	1.019	1.020	0.857	0.890	0.818	0.753	0.721	0.890
	Hebei	1.033	1.053	1.034	1.037	1.028	0.790	0.696	0.611	0.574	0.873
	Liaoning	0.519	0.501	0.527	0.532	0.529	0.562	0.516	0.462	0.385	0.504
	Shanghai	1.487	1.460	1.462	1.464	1.525	1.466	1.386	1.297	1.231	1.420
	Jiangsu	0.883	0.901	0.925	1.001	1.008	1.047	1.024	1.011	0.930	0.970
	Zhejiang	1.147	1.110	1.124	1.168	1.163	1.190	1.213	1.250	1.346	1.190
	Fujian	0.795	0.727	0.725	0.732	0.728	0.715	0.729	0.727	0.712	0.732
	Shandong	0.886	0.793	0.813	0.823	0.860	0.852	0.828	0.743	0.752	0.817
	Guangdong	1.086	1.113	1.114	1.097	1.091	1.025	1.011	0.957	0.860	1.039
	Hainan	0.635	0.667	0.678	0.683	0.679	0.647	0.609	0.570	0.542	0.634
Central	Shanxi	0.413	0.405	0.407	0.401	0.414	0.432	0.396	0.356	0.329	0.395
	Jilin	0.461	0.430	0.420	0.401	0.407	0.441	0.456	0.345	0.281	0.405
	Heilongjiang	0.514	0.539	0.548	0.566	0.611	0.577	0.460	0.383	0.298	0.500
	Anhui	1.001	1.047	1.041	1.029	1.002	1.019	1.013	0.890	0.849	0.988
	Jiangxi	0.954	1.033	1.074	1.107	1.101	1.101	1.104	1.042	1.016	1.059
	Henan	1.016	1.003	1.024	1.025	1.038	1.047	1.033	1.015	1.022	1.025
	Hubei	0.911	0.881	0.939	0.968	1.015	0.995	1.006	1.010	0.890	0.957
	Hunan	0.721	0.764	0.851	1.001	1.008	0.850	1.010	0.887	1.012	0.900
West	Inner Mongolia	0.440	0.445	0.434	0.436	0.454	0.452	0.423	0.338	0.340	0.418
	Chongqing	1.076	1.030	1.116	1.140	1.134	1.181	1.106	1.112	1.118	1.113
	Guangxi	0.939	1.096	1.064	1.043	1.033	0.907	0.902	1.016	1.018	1.002
	Sichuan	0.892	0.834	0.881	0.904	0.922	0.908	0.909	0.957	1.004	0.912
	Guizhou	1.265	1.143	1.033	0.813	0.740	0.711	0.770	0.785	0.682	0.882
	Yunnan	1.043	1.032	1.020	1.016	1.023	0.903	0.925	0.883	0.902	0.972
	Tibet	0.485	0.420	0.415	0.414	0.441	0.493	0.428	0.349	0.350	0.422
	Shaanxi	0.567	0.556	0.563	0.577	0.581	0.605	0.611	0.542	0.442	0.560
	Gansu	0.612	0.622	0.610	0.643	0.694	0.698	0.689	0.647	0.612	0.647
	Ningxia	0.717	0.681	0.693	0.679	0.665	0.661	0.597	0.547	0.541	0.642
	Qinghai	0.603	0.553	0.528	0.478	0.530	0.540	0.470	0.459	0.459	0.513
	Xinjiang	0.590	0.612	0.715	0.800	0.802	0.732	0.671	0.669	0.571	0.685
	Average	0.830	0.826	0.836	0.842	0.845	0.825	0.805	0.767	0.738	0.813

### 3.3. Productivity changes

#### 3.3.1. Productivity change across provinces

Table 5 displays the average TFP in the 31 provinces from 2012 to 2020. Only the values in Beijing and Zhejiang were greater than 1, while the remainder showed different degrees of decline, suggesting that efficiency decreased broadly during this time. Significant differences were observed between provinces, with Zhejiang experiencing the most growth and Guizhou the largest decline, of 10.5%. As Zhejiang and Beijing attract competent health technicians by offering competitive salaries and supplying advanced equipment, patients are likely inclined to prefer facilities in these two cities, further improving their competence in treatment and management.

**Table 5:** Malmquist index and decomposition indexes by province.

	Province	PEC	SEC	TC	EC	TFP
East	Beijing	1.004	0.995	1.003	0.999	1.002
	Tianjin	1.000	0.975	0.986	0.975	0.961
	Hebei	0.933	1.000	0.960	0.933	0.896
	Liaoning	0.974	0.989	0.970	0.963	0.934
	Shanghai	0.980	0.997	0.990	0.977	0.968
	Jiangsu	1.015	0.993	0.980	1.008	0.988
	Zhejiang	1.050	0.972	0.997	1.020	1.016
	Fujian	0.990	0.997	0.969	0.987	0.956
	Shandong	1.009	0.971	0.969	0.980	0.950
	Guangdong	0.983	0.988	0.986	0.971	0.958
	Hainan	1.000	0.982	0.982	0.982	0.964
	Mean	0.994	0.987	0.981	0.981	0.963
Central	Shanxi	0.986	0.987	0.969	0.973	0.943
	Jilin	0.963	0.976	0.979	0.940	0.920
	Heilongjiang	0.958	0.975	0.971	0.934	0.907
	Anhui	0.995	0.987	0.966	0.982	0.949
	Jiangxi	1.003	1.006	0.951	1.009	0.960
	Henan	1.009	0.992	0.967	1.000	0.967
	Hubei	1.007	0.990	0.973	0.997	0.970
	Hunan	1.040	1.003	0.928	1.043	0.968
	Mean	0.995	0.990	0.963	0.985	0.948
West	Inner Mongolia	0.987	0.983	0.973	0.971	0.944
	Chongqing	1.007	0.999	0.978	1.006	0.983
	Guangxi	1.010	1.004	0.959	1.014	0.973
	Sichuan	0.982	1.034	0.951	1.015	0.965
	Guizhou	0.931	0.998	0.962	0.930	0.895
	Yunnan	0.984	0.999	0.968	0.983	0.951
	Tibet	0.986	0.979	0.980	0.965	0.945
	Shaanxi	0.978	0.994	0.973	0.973	0.947
	Gansu	1.008	0.993	0.973	1.000	0.973
	Qinghai	1.005	0.964	0.965	0.969	0.935
	Ningxia	0.993	0.973	0.989	0.966	0.955
	Xinjiang	1.006	0.994	0.963	1.000	0.963
	Mean	0.990	0.993	0.970	0.983	0.952

#### 3.3.2. Productivity change across regions

As can be seen from Table 6, the efficiency in the eastern region declined the least, mainly due to having the smallest decline in TC, and the central region declined the most, by 5.2%, which is contrary to the findings of Chen et al. that TFP growth was higher in the interior provinces than in the western region (37). Despite slower economic development, a level of medical resource reserves has been accumulated in recent years in the western region from perennial national counterpart assistance and policy preferences. However, the central region has neither developed to the same economic level as the eastern region nor received the strong policy and counterpart support of the western region, and due to the proximity of the eastern region, where health technicians are paid more, the central region suffers from a greater loss of skilled health technicians than the western region. Consequently, the central region’s Malmquist index is lower than that of the western region.

**Table 6:** Malmquist index and decomposition indexes by region.

Region	PEC	SEC	TC	TEC	TFP
East	0.994	0.987	0.981	0.981	0.962
Central	0.995	0.990	0.963	0.985	0.949
West	0.990	0.993	0.970	0.983	0.954

#### 3.3.3. Productivity change over time

Table 7 shows the annual average TFP and its components from 2012 to 2020. Despite fluctuations in some years, there is an overall downward trend. The average TFP from 2012 to 2020 was 0.955, and apart from 2012 to 2013, all the other years displayed a downward trend, indicating that PHC services at the provincial level declined in productivity over time. TFP in 2019–2020 had the largest decline, of 17%, which was mainly due to the decline in TC of 12.4%. The decomposition index shows a downward trend for PEC, SEC, TC and TEC, suggesting the level of technology and management of PHCIs is still limited, producing a decline in efficiency.

**Table 7:** Malmquist index and decomposition indexes by year.

Year	PEC	SEC	TC	TEC	TFP
2012–2013	1.005	0.990	1.027	0.994	1.012
2013–2014	1.012	1.002	0.962	1.014	0.975
2014–2015	0.999	1.008	0.956	1.007	0.963
2015–2016	1.016	0.993	0.986	1.009	0.995
2016–2017	0.982	1.003	1.005	0.985	0.989
2017–2018	0.990	0.975	0.965	0.966	0.932
2018–2019	0.968	0.974	1.000	0.942	0.942
2019–2020	0.972	0.975	0.876	0.948	0.830
Average	0.993	0.990	0.972	0.983	0.955

**Figure 3:**
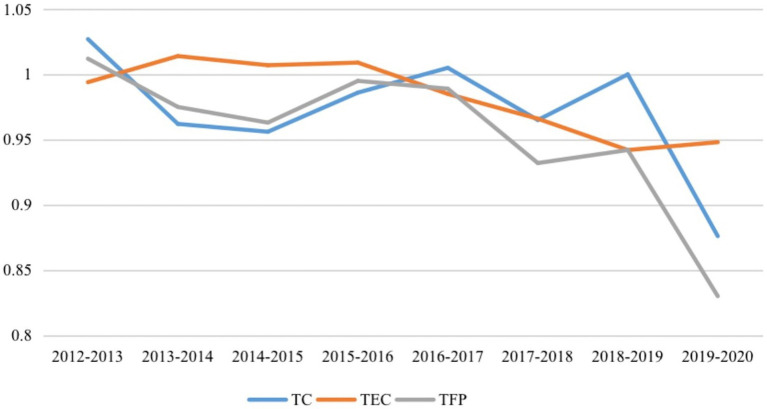
TC, TEC and TFP averages of the Malmquist Index by year.

Combined with Figure 3, it can be seen that the TC trend essentially tracks that of TFP. Pearson’s correlation coefficient for TC and TFP over time is 0.889 (*p* = 0.003), indicating that there is a significant and strong correlation between TC and TFP, which further illustrates that TC is the main factor affecting the productivity change. This is consistent with other studies that have found similar patterns in TFP. For instance, health system productivity in China was compared before and after the 2009 health care reforms, and the results showed that the observed decline was mostly attributable to the deceleration in TC (32). Similarly, Ng (38) found that China’s health care systems were experiencing productivity decline, which was linked to technological regression.

### 3.4. Determinants of technical efficiency

While an estimation of efficiency is an examination of the effectiveness of past reforms, an exploration of the determinants of efficiency is even more important for the government to adjust the future direction of health care reform and to develop effective policies. As shown in Table 8, economic development affects the efficiency of PHC services, which can explain the distribution in Figure 2. Urbanization and education also have an impact on efficiency, which is consistent with previous studies (39).

**Table 8:** Tobit regression results.

	Name	Model 1	Model 2	Model 3
Independent variables	Financial support	0.326** (0.081)	–	0.397** (0.065)
	Social health insurance coverage	–	−0.079** (0.04)	−0.148* (0.051)
Control variables	Population density	0.026 (0.022)	0.03 (0.025)	0.017 (0.025)
	Urbanization	0.234** (0.028)	0.281** (0.025)	0.290** (0.036)
	Education	−0.073** (0.031)	−0.217** (0.026)	−0.083** (0.028)
	GDP *per capita*	0.497** (0.079)	0.449** (0.024)	0.606** (0.052)
	Constant	0.144** (0.068)	0.364** (0.027)	0.165* (0.065)

*p < 0.05; **p < 0.01.

As shown in Model 1, financial support significantly improves the efficiency of PHC services, which supports our hypothesis. This observation is consistent with Evans et al. (4), who found that efficiency is positively associated with health expenditure *per capita*. Similarly, a study conducted in Shandong province found that basic public health services operated more effectively once the proportion of total public expenditures spent on health was increased (40), which is not surprising because a proper expenditure arrangement entails increased access to and use of health resources by the public. Currently, most patients still view PHCIs as locations for dispensing and refilling prescriptions rather than for treatment and consultation, and most patients still favor secondary or tertiary facilities when seeking care due to a lack of skilled health technicians and equipment (41). An increased share of public budgets allocated to health can help to improve the technical conditions and personnel quality of health services, which would improve the operating conditions and service levels of PHCIs, making it easier to retain experienced health technicians and improving technology, which would together contribute to increased efficiency and attract more patients.

As noted in Model 2, social health insurance is negatively correlated with the efficiency of PHC services, which contradicts our hypothesis. Several studies confirmed our findings. The increase in social health insurance coverage may not have a major impact on the efficiency for the reason of market power and moral hazard effect (42). Meanwhile, it will promote the use of high quality of health care services, which affected the utilization of PHC, resulting in inefficiencies. From a practical point of view in China, it may be a consequence of three different factors. First is poor reimbursement structures (43): social health insurance, especially the NRCMS, does little to address the issues of unaffordable access to health care due to high deductibles and co-payments, poor reimbursement rates and convoluted reimbursement procedures (44), and low reimbursement caps substantially increase patients’ financial risk and restrict their service options. After annual caps are met, patients with minor health conditions will seek outpatient care at secondary and tertiary hospitals, especially if the reimbursement caps in hospitals are higher than those in PHCIs (45), which lead to the low utilization of facilities in PHC. Meanwhile, NRCMS prioritizes inpatient treatments above outpatient services, and outpatient service reimbursement is low (46). Expensive outpatient services therefore remain a heavy financial burden for many patients, such that outpatient utilization has barely increased, which is detrimental to efficiency.

The second factor may be inappropriate payment methods (47): at present, the dominant health insurance payment method in China is fee-for-service, which, as a type of retrospective payment system, is considered harmful to efficiency, causing health care providers to work inefficiently or seek to induce demand (48). Fee-for-service also leads to a pattern of competition for patients at all levels of health care in order to increase the volume of visits. PHCIs are clearly unable to compete with secondary and tertiary hospitals in terms of technology and resources, so more patients are absorbed by public hospitals, resulting in low utilization of PHC services, which hinders efficiency improvements.

The third factor may be incomplete support policies: the National Essential Medicines System (NEMS), which mandates that only essential medicines can be used by PHCIs, is closely related to the social health insurance policy, according to which the breadth and capacity of PHCIs to make diagnoses and provide medical care is constrained, which in turn hampers the growth of medical specialties and competencies. A shortage of medicines would also force people to seek care at public hospitals, which would further hinder efforts to improve the efficiency of PHC services. In Model 3, we combined financial support with social health insurance coverage, and they both remained significant, confirming their effects on efficiency.

## 4. Discussion and conclusion

Improving healthcare efficiency has drawn significant attention for many years, especially in developing countries. By estimating efficiency, health planners and decision-makers can identify bottlenecks and adopt solutions in line with national health goals, and given the many similarities among developing countries, the findings of this China-focused study may serve as a reference. The results demonstrate the inefficiency of PHC services in China and show obvious regional differences, with the highest efficiency in the east. Productivity also shows a downward trend over time, mainly due to a decline in TC. Financial support can improve the efficiency of PHC services, but social health insurance coverage—at least, as it is currently implemented in China—decreases efficiency.

The decomposition of the Malmquist Index shows that the common factor in the decline in productivity in the three regions is the deterioration in TC, which implies that progress and technological innovation are key factors for efficiency that help to attract patients to visit PHCIs by improving service capacity. Although medical resource reserves have been accumulated in recent years from perennial national investment and policies, it is also critical to improve the quality of such resources, especially medical technology, health technicians and management support. More competent general practitioners should be trained, and the salaries and welfare benefits of health technicians should be improved (49). However, this would entail a long and gradual process to achieve productivity improvement, especially for the central and western regions.

Our findings confirm that efficiency in China varies between the three regions, which correlates with socio-economic factors, including economic development, urbanization and education. Higher economic development is more likely to lead to the adoption of a range of innovative measures, including comprehensive infrastructure, and a population with more education will tend to be knowledgeable and make better health choices. A higher level of urbanization also makes medical institutions more concentrated and thus makes it easier to exploit economies of scale and to improve technical and service efficiency through competition, technology spill-over and diffusion. In a word, higher level of urbanization and economic development means higher income, better infrastructure and better supply of health care services, which contribute to the promotion of the efficiency (50). In contrast, low economic development, education and urbanization in the central and western regions hinder efficiency, but policy alone cannot easily act upon these determinants of efficiency. Nevertheless, our findings suggest that, as there are such major differences between the regions, the design and execution of measures should be tailored to each and more resource, and subsidy support should be provided to the central and western regions to narrow the gap with the economically developed east.

Financial support is also beneficial for efficiency, suggesting that developing countries may need to increase the share of the public budget devoted to health care because there remains a large gap between developing and developed countries in terms of financial support for their health care systems. Low expenditure results in the under-provision of public services and a high private share of total health expenditure, which increases costs to patients due to problems of geographic access, availability and financial accessibility (51); conversely, increasing expenditure would enable PHCIs to purchase advanced equipment and hire experienced health technicians, improving the quality of PHCIs and attracting patients for treatment. Nevertheless, we cannot ignore that limitations on public funds make it difficult to achieve sustained increases in health expenditure, and it is therefore equally important to improve productivity and efficiency in the health systems of developing countries (52).

To achieve universal coverage, many developing countries are considering increased investment in their national insurance programmes. We cannot discount the advantages of social health insurance for ensuring access to health care services and for protecting households from financial risk caused by the cost of health care services, but the success of social health insurance may be limited. The multiple regression results indicate that insurance coverage generates inefficiency. Reasonable reimbursement design, appropriate payment methods and comprehensive support policies are a key step to achieving universal coverage, not merely investing more in national insurance schemes. As Bloom (53) said, any health care reform programme eventually needs to address the underlying institutional arrangements inherent in the system, and a crucial issue is how to better implement a policy of differential payment of medical insurance for different levels of health care institutions and thus raise the proportion of medical insurance payments for PHCIs. The public is increasingly concerned about the quality of health care services, but financial protection also remains a concern, especially among those with low incomes. In terms of payment methods, the Chinese government should consider a more varied approach than payment by service item, such as diagnosis-related groups (DRGs), capitation, case-mix or global budgets. NEMS support policies should also be improved, and increasing reimbursement rates for—and expanding the range of—essential medicines should be considered.

Compared to the studies on health care efficiency, increasing financial support and enhancing the relative attention of the government remains an important policy tool for efficiency improvement, which is consistent with most findings. In the case of health care insurance, however, it is noted that there is no impact on improvement of PHC efficiency, which has significant implications for policy makers in the future. For developing countries, PHC reform is moving into the ‘deep water zone’ phase and is still a long way from achieving universal health coverage. Restoring and improving PHC efficiency will be an incremental process, and COVID-19 placed the PHC system under tremendous pressure in terms of performance, sustainability and quality. It is therefore crucial for developing countries to explore new methods to improve productivity and efficiency in their health systems. We plan to continue our focus on the efficiency of PHC services, and we encourage other researchers to engage in empirical research on this topic in developing countries in order to extend our analysis.

## Data availability statement

Publicly available datasets were analyzed in this study. This data can be found here: http://www.stats.gov.cn.

## Author contributions

ZZ, SD, JW, and QJ contributed to the conception and design of the study. ZZ wrote th first draft of the manuscript. ZZ and SD organized the database and performed the statistical analysis. JW and QJ reviewed and edited the article. All authors contributed to the article and approved the submitted version.

## Funding

Project research on improving the accessibility of public services in rural areas from the perspective of policy tools supported by the Fundamental Research Funds for the Central Universities (Grant no. 2662023GGPY002).

## Conflict of interest

The authors declare that the research was conducted in the absence of any commercial or financial relationships that could be construed as a potential conflict of interest.

## Publisher’s note

All claims expressed in this article are solely those of the authors and do not necessarily represent those of their affiliated organizations, or those of the publisher, the editors and the reviewers. Any product that may be evaluated in this article, or claim that may be made by its manufacturer, is not guaranteed or endorsed by the publisher.
